# Pyeloplasty and Ureteral Reconstruction Surgery Trends: A Total Population Analysis in Germany from 2006 to 2022

**DOI:** 10.1016/j.euros.2024.10.011

**Published:** 2024-10-24

**Authors:** Luka Flegar, Felix Kipfer, Tufan Durmus, Nicole Eisenmenger, Philipp Karschuck, Cem Aksoy, Philipp Reimold, Thomas Martin, Lennard Haak, Rainer Koch, Rudolf Moritz, Johannes Huber, Christer Groeben

**Affiliations:** a Department of Urology, Philipps-University Marburg, Marburg, Germany; b Department of Urology, Royal Melbourne Hospital, Melbourne, VIC, Australia; c Reimbursement Institute, Hürth, Germany; d Department of Urology Marien Hospital Herne, University Hospital of the Ruhr-University of Bochum, Bochum, Germany

**Keywords:** Pyeloplasty, Minimally invasive surgery, Health services research, Robotics, Population-based analysis

## Abstract

**Background and objective:**

With advancement in technology, it has been possible to use minimally invasive surgical approaches for performing pyeloplasty for the treatment of ureteral strictures. This study aims to investigate the current trends of pyeloplasty and reconstructive ureteral procedures.

**Methods:**

We analyzed the nationwide German hospital billing database (Destatis) from 2006 to 2022. Linear regression models were utilized for the identification of trends over time.

**Key findings and limitations:**

A total of 34 975 pyeloplasties and 37 470 cases with ureteral reconstruction procedures were analyzed. The total number of pyeloplasties increased from 1990 procedures in 2006 to 2251 in 2019, before declining again down to 1916 procedures in 2022 (*p* = 0.783). Open pyeloplasty cases decreased from 79.5% in 2006 to 17.6% in 2022, while those using the robotic approach increased from 0.3% in 2006 to 35.9% in 2022 (*p* < 0.001). The median length of stay (LOS) for open pyeloplasty decreased from 13 d in 2006 to 8 d in 2022 (*p* < 0.001). The median LOS for robotic pyeloplasty decreased from 8 d in 2008 to 5 d in 2022 (*p* < 0.001). In-hospital mortality was 0.15% after open pyeloplasty versus 0.07% for robotic-assisted pyeloplasty versus 0.03 for laparoscopic pyeloplasty (*p* = 0.009 for comparison of open vs robotic). Reconstructive ureteral surgical cases per year appeared relatively stable, with 1929 cases in 2006 and 2014 cases in 2022 (*p* = 0.713). A surgical robot was used in 4.5% of all cases with a ureter reconstruction, with inclining shares per year from 0.2% in 2009 and 18.3% in 2022 (*p* < 0.001).

**Conclusions and clinical implications:**

This study showed an increasing trend toward minimally invasive pyeloplasty in recent years. For reconstructive procedures, the share of robotics was less pronounced. LOS decreased for all procedures and was shortest for the robotic approach.

**Patient summary:**

In this study, current trends of pyeloplasty and reconstructive ureteral procedures in Germany between 2006 and 2022 were investigated. In recent years, minimally invasive pyeloplasty is increasingly being used and length of hospital stay has also decreased.

## Take Home Message

Our study investigated trends in pyeloplasty and reconstructive procedures of the ureter in Germany between 2006 and 2022. We noticed an increase in the use of robotic-assisted procedures, especially for pyeloplasty. The length of hospital stay decreased in recent years.

## 1 Introduction

Strictures or other obstructions of the ureter are a rare cause for patients to consult their urologist or a urological outpatient department [1]. The causes can be diverse and thus require individual surgical treatment concepts accordingly. A relevant share of cases of ureteral strictures are generated by ureteral injury during endoscopic treatment for renal or ureteral stones or tumors [2], [3]. Other frequent iatrogenic causes are pelvic surgery or pelvic and retroperitoneal radiotherapy [4], [5]. Rare cases can be caused by endometriosis or ureteral tumors.

The main surgical goal of treating ureteral dysfunction is to restore a nonobstructive urine passage and thus preserve renal function [6]. There are numerous treatment concepts for this purpose, from endoscopic incisions and stenting to partial or complete replacement of the ureter with autologous or nonautologous material and percutaneous urinary diversion [7], [8]. The localization as well as the length of the stricture and the patient's fitness for surgical intervention are decisive for the selection of the appropriate therapy. The most proximal stenosis of the ureter is the ureteropelvic junction obstruction. This mostly congenital malformation is usually treated by resection of the narrowed ureteral segment and the dilated portion of the renal pelvis, and reanastomosis of the renal pelvis to the remaining ureter commonly known as pyeloplasty [9].

Strictures of the proximal or middle ureter can be treated by resection of the narrowed section in combination with a reanastomosis of both ends of the ureter in the case of short-segment stricture. If the constricted section is more extended longitudinally, reconstruction is required [8]. Various sections of the gastrointestinal tract can be applied, with the ileum interposition being the most commonly used option [10], [11], [12]. However, strictured segments of the distal ureter are oftentimes replaced by an elevation or a mobilized flap of the bladder with a following ureterocystoneostomy.

Traditionally, the abovementioned procedures, especially those using sections of the gastrointestinal tract, were performed with a laparotomy [8], [13]. Owing to advances in the technological development of endoscopy and, most recently, robotics, minimally invasive surgical approaches have become possible [14], [15]. For selected indications (eg, pyeloplasty) these surgical techniques could have the potential of becoming the new standard [9].

This study is intended to assess the current trends and treatment patterns in the surgical treatment of ureteral strictures as well as of the applied reconstructional procedures in Germany in the past two decades. Special focus is placed on the use of surgical approach and the differences in the length of stay (LOS).

## 2 Patients and methods

Data from the Federal Statistical Office of Germany (Destatis) were analyzed. The Destatis database was assessed to examine all surgical procedures and associated diagnoses. The techniques for extracting data and identifying cohorts were described in detail in previous studies [16]. Supplementary Table 1 provides an overview of the queried database.

### 2.1 Federal Statistical Office of Germany (Destatis)

The data are coded using the International Classification of Disease Modification 10 (ICD-10) coding system for diagnosis and “Operationen und Prozedurenschluessel” (OPS; German version of the international classification of procedures in medicine) corresponding to the interventions conducted. The inclusion criteria were age of at least 18 yr combined with the following codes for interventions. For the present analysis, we used the following OPS codes: 5-557.4—pyeloplasty, 5-568.1—ureter reanastomosis, 5-568.b and 5-568.d—ureterocystoneostomy, and 5-568.g—ileal ureter replacement, along with the respective subcodes for surgical approach (open, laparoscopic, and 5-987 for robotic). In addition, we analyzed the combination of OPS codes 5-242.6 (removal of a mucosal graft from the cheek), 5-568 (reconstruction of ureter), and 5-584.7 and 5-584.8 (reconstruction of the urethra using a buccal graft) according to the coding practice of some centers.

### 2.2 Statistical analysis

Data are presented using absolute and relative frequencies. For the identification of trends over time, linear regression models were utilized. For calculations, chi-square test was applied. Statistical significance was defined as *p* < 0.05. For statistical analysis, SAS 9.4 (SAS Institute GmbH, Heidelberg, Germany) was used.

## 3 Results

**Fig. 1:**
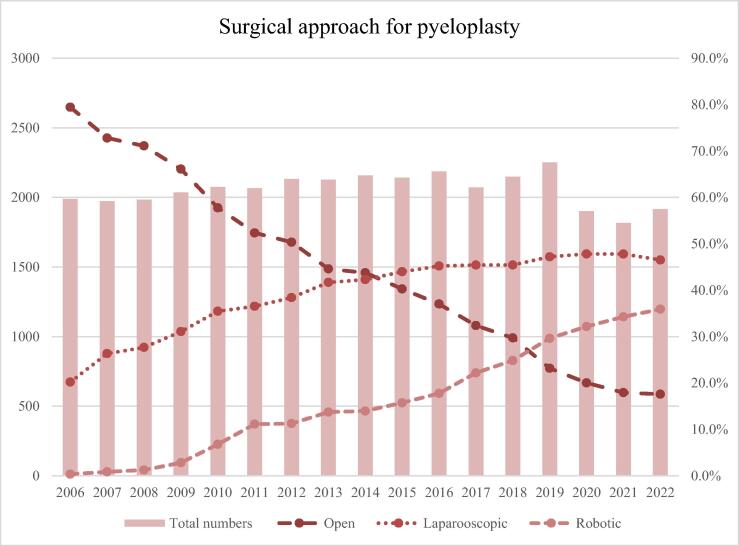
Yearly pyeloplasty procedures from 2006 to 2022 (.

source: Destatis). Dotted lines display the shares of surgical approach (right vertical axis), while pillars show the total caseload (left vertical axis)

In total, 34 975 pyeloplasties were analyzed. The total number increased from 1990 procedures in 2006 to 2251 in 2019 before declining again down to 1916 procedures in 2022 (*p* = 0.783 for the overall trend). Open pyeloplasty cases decreased from 79.5% in 2006 to 17.6% in 2022, while cases using the robotic approach increased from 0.3% in 2006 to 35.9% in 2022 (*p* < 0.001; Fig. 1). Laparoscopic pyeloplasties increased from 20.2% in 2006 to 46.5% in 2022 (*p* < 0.001). The case numbers for pyeloplasty were reduced by 12.9% during the last years (2020–2022), compared with the previous 3 yr (2017–2019). However, this was true only for the open and laparoscopic approaches, while caseload increased for the robotic approach by 29.9% during the last years (*p* < 0.001).

**Fig. 2:**
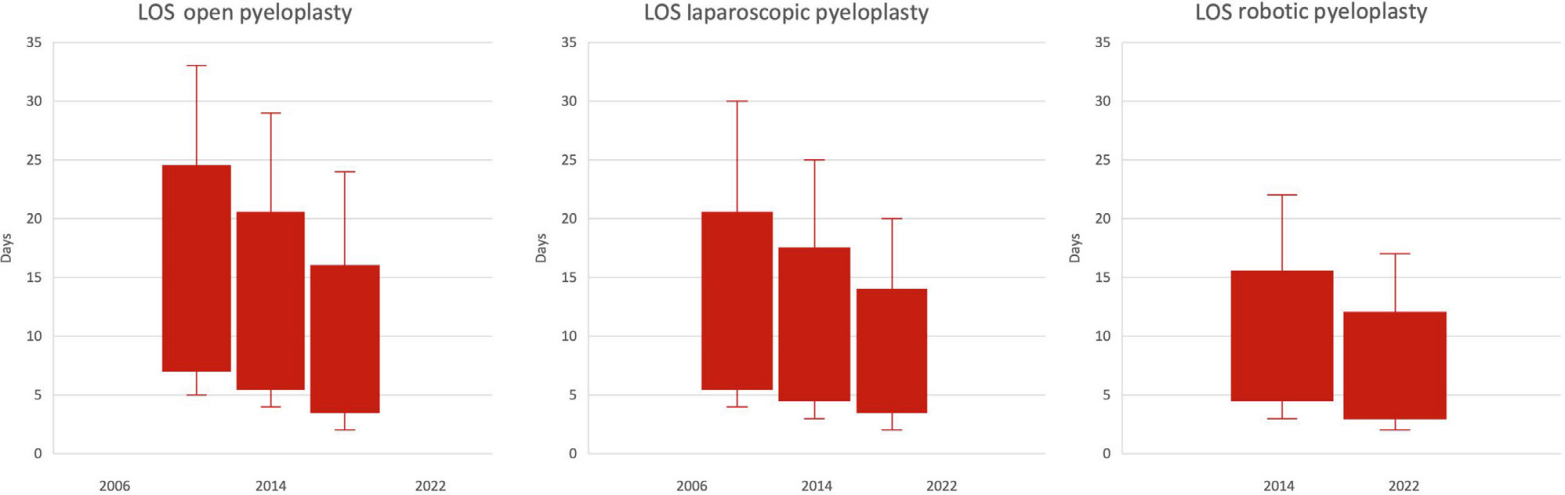
Length of stay for open versus laparoscopic versus robotic pyeloplasty (.

source: Destatis). LOS = length of hospital stay

Figure 2 displays the LOS for open versus laparoscopic versus robotic-assisted pyeloplasty. The median LOS for open pyeloplasty decreased from 13 d in 2006 to 8 d in 2022 (*p* < 0.001). The median LOS for laparoscopic pyeloplasty decreased from 9 d in 2006 to 7 d in 2022 (*p* < 0.001). The median LOS for robotic pyeloplasty decreased from 8 d in 2008 to 5 d in 2022 (*p* < 0.001).

In-hospital mortality was 0.15% after open pyeloplasty versus 0.07% for robotic-assisted pyeloplasty versus 0.03 for laparoscopic pyeloplasty (*p* = 0.009 for comparison of open vs robotic pyeloplasty).

In 2006, 351 hospitals performed pyeloplasty in Germany, which remained fairly stable up to 379 hospitals in 2022 (*p* = 0.965). In 2006, six out of 351 hospitals performed >20 procedures (high caseload) per year, while it increased to 15 out of 413 in 2014. In 2022, ten out of 379 hospitals performed >20 procedures per year.

Of the patients undergoing pyeloplasty in 2006, 68.8% were older than 18 yr, while it increased to 70.1% in 2020. The mean age for adult patients for pyeloplasty was 48.7 ± 18.3 yr, while patients for the open approach were older (age 50.7 ± 18.2 yr) than those for robotic pyeloplasty (age 47.3 ± 18.2 yr; *p* < 0.001) and laparoscopic pyeloplasty (age 46.2 ± 18.0 yr; *p* < 0.001). The mean age increased for all pyeloplasty procedures from 46.4 ± 18.0 yr in 2006 to 51.0 ± 18.0 yr in 2022 (*p* < 0.001; Supplementary Fig. 1).

**Fig. 3:**
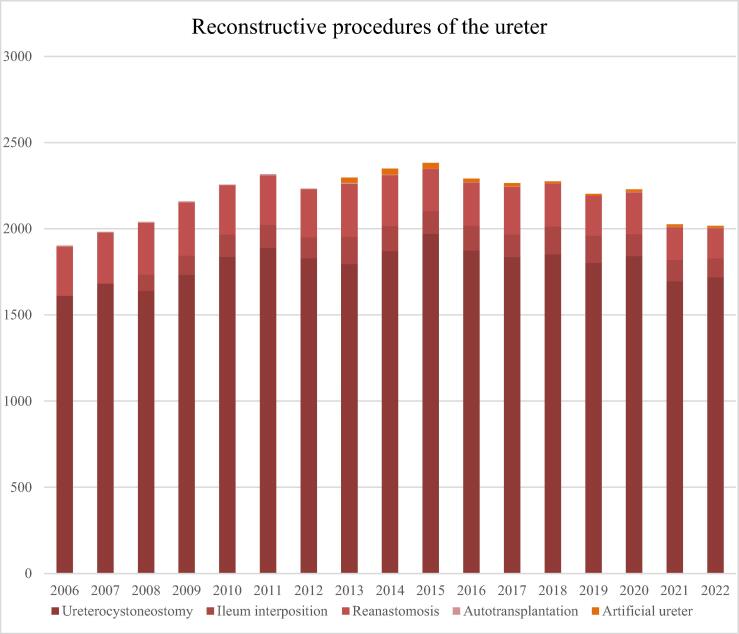
Total numbers of reconstructive procedures of the ureter from 2006 to 2022 (.

source: Destatis)

We were able to include a total of 37 470 cases with reconstruction of the ureter. The number of cases per year appeared relatively stable, with 1929 cases in 2006 and 2014 cases in 2022 (*p* = 0.713 for trend). Reanastomosis could be performed in 4501 cases (12.0%). An unspecified partial ureteral replacement was performed in 9862 cases (26.3%), an unspecified total ureter replacement was performed in 509 cases (1.4%), a ureterocystoneostomy was performed in 20 618 cases (55.0%), and a ureteral replacement using bowel segments was performed in 1980 cases (5.3%; Fig. 3). Between 2006 and 2022, reconstruction of the ureter with a buccal graft was coded in 38 cases, with 22 cases (62.9%) being performed in the last years (2020–2022). Procedures of ureterocystoneostomy increased slightly from 1019 cases in 2006 to 1277 cases in 2019 (*p* < 0.001) and bowel interposition increased from 93 cases in 2008 to 158 cases in 2019 (*p* = 0.003). Sixty-seven cases of autotransplantation for reconstruction of the ureter were performed during the analyzed period, which decreased from six cases in 2006 to three cases in 2022, while the cases with implantation of an artificial ureter (Detour-System) decreased from 30 cases in 2013 to ten cases in 2022 (*p* < 0.001). All case numbers were reduced slightly (by 7.2%) during the last years (2020–2022) compared with the previous 3 yr (2017–2019), with the most reduction of caseload occurring for total ureter replacement (31.8%), usage of bowel for reconstruction (20.6%), and reanastomosis of the ureter (19.7%). A surgical robot was used in 1679 cases with a ureter reconstruction (4.5%), with inclining shares from 0.2% in 2009 and 18.3% in 2022 (*p* < 0.001). Although coding for conventional laparoscopic ureter reconstruction was possible, no cases were recorded during the investigated period of time.

The median LOS for ureter reconstruction using reanastomosis amounted to 16 d overall, with a declining trend from 20 d in 2006 to 14 d in 2022 (*p* < 0.001). The LOS decreased from 17 d (2006) to 11 d (2022) for partial ureter replacement, from 20 d (2006) to 13 d (2022) for total ureter replacement, from 19 d (2006) to 11 d (2022) for ureterocystoneostomy, and from 21 d (2006) to 14 d (2022) for bowel interposition (*p* < 0.001 for all trends).

## 4 Discussion

The present population-based study investigates current trends in pyeloplasty as well as ureter reconstruction procedures in Germany. Several main observations were made: while the number of pyeloplasty cases increased only slightly over the past two decades, the surgical approach has changed significantly. In 2006, open pyeloplasty was the predominant method, but by 2022, minimally invasive pyeloplasty was used in four out of five patients. Additionally, LOS for pyeloplasty has decreased significantly in recent years across all surgical approaches. In contrary, we noticed a low proportion of robotic surgeries for reconstructive ureteral procedures, while the total number of ureteral reconstruction surgeries remained stable during the investigated period and decreased slightly due to the coronavirus disease 2019 (COVID-19) pandemic within the last 3 yr of our analysis.

### 4.1 Pyeloplasty

Our results showed that pyeloplasty cases increased slightly between 2006 and 2019, with the highest share of increase for the robotic approach. While open and laparoscopic pyeloplasty decreased in the recent time, the robotic approach increased by 29.9% in the last 3 yr of our investigation. Robotic pyeloplasty is generally less traumatizing than the open approach and therefore associated with a faster recovery. Hopf et al [17] investigated long-term outcomes for robotic pyeloplasty in patients with ureteropelvic junction obstruction. The authors were able to show excellent results with an 8-yr failure-free survival rate of 96.3%. A recent study reported a similar success rate of robotic pyeloplasty to that of open pyeloplasty, with lower intraoperative blood loss and lower rates of early postoperative complications [18]. Additional benefits include improved cosmetic results. The authors also contend that the higher costs associated with the robotic approach will decrease in the future due to the introduction of new, less expensive robotic platforms. Another study compared complications and outcomes between laparoscopic and robotic pyeloplasty, showing comparable results overall [19].

In the present analysis, in-hospital mortality was low after pyeloplasty, indicating the safety of the procedure. Similar results were shown by a national study from the UK regarding the practice and outcomes of pyeloplasty [20].

Median LOS decreased significantly for all surgical approaches. LOS for robotic pyeloplasty decreased to 5 d in 2022, making it the shortest among all surgical approaches. Similar findings were reported by Braga et al [21]. The observed decrease in LOS for both open and minimally invasive approaches can be explained with the increasing implementation of enhanced recovery after surgery (ERAS) protocols. ERAS protocols are designed to reduce postoperative stress, preserve perioperative physiological functions, and promote early mobilization [22]. Another factor possibly affecting LOS could be the learning curves of the respective urological surgeons. Several studies have shown that as surgeons advance along the learning curve, their surgical efficiency improves, leading to reduced operative times and potentially shorter LOS for patients [23].

During the analysis of hospitals performing pyeloplasty in Germany, we noticed a slightly increasing trend toward high-caseload centers, with ten out of 379 hospitals performing >20 procedures per year in 2022. Our working group reported comparable results for other surgical procedures, for example, radical cystectomy or kidney transplantation [16], [24].

Interestingly, during our analysis, the mean age increased for all pyeloplasty procedures from 46.4 ± 18.0 yr in 2006 to 51.0 ± 18.0 yr in 2022. Patients undergoing open pyeloplasty were older than those undergoing minimally invasive approaches, although several studies reported good outcomes for minimally invasive pyeloplasty of elderly patients [25].

### 4.2 Reconstruction

In our analysis, we saw a relatively stable trend in the total number of ureteral reconstruction cases over the 15-yr period, with the exception of the last 2 yr during the COVID-19 pandemic. Here, there was a brief decline in case numbers of around 7%, which continued until 2022. As ureteral reconstructions are lower priority procedures than, for example, oncological procedures, which can also be bridged easily, for example, using ureteral stents, this seems understandable in view of the general pandemic precautionary measures, including the reduction in surgical capacity for elective procedures [26]. This is also reflected in analyses and reports by other colleagues from similar entities in other European countries [27]. On the contrary, it seems surprising that the extent of case reduction was rather low at <10% despite the temporary considerable restrictions on surgical operations in the respective clinics and large medical care centers [28]. Ureteral reconstructions may be required in two fundamentally different situations. An indication for this procedure exists if, for example, due to anatomical, post-traumatic, or iatrogenic reasons, a secondary ureteral stricture or lesion develops, and free urine flow is no longer ensured [2], [3]. In such cases, it is possible to bridge the stricture with a ureteral stent or nephrostomy, and plan the procedure secondarily [7], [8]. However, it is also possible that during an abdominal, retroperitoneal, or pelvic surgery, an acute ureteral injury of such severity occurs that immediate reconstruction is necessary [8]. Owing to the underlying data structure and the given national anonymization regulations, these two fundamentally different initial situations cannot be separated from each other in ureteral reconstructions. Thus, reconstructions after iatrogenic lesion of the ureter, which could not be postponed, might have mitigated the decline of case numbers during the pandemic. Furthermore, the higher decline in case numbers for pyeloplasty than for ureteral reconstructions could be explained by the fact that the need for immediate reconstruction is significantly less frequent in pyeloplasty [6], [9].

As expected, robotic surgery for reconstructive ureteral procedures began to expand, albeit initially hesitantly but continuously, starting in the mid-2000s. This has also been demonstrated by our research group for other entities and corresponding surgical procedures [29]. Notably, the relatively low proportion of robotic surgeries up to the year 2022, even in comparison with pyeloplasty (18% vs 35%), is noticeable. This could be explained by two important aspects. First, pyeloplasty, at least in the primary situation, is a procedure that is largely standardized and generally of lower difficulty [6], [9]. This could lead to a quicker adoption as a “beginner's procedure” for novice robotic surgeons. Second, the need for ureteral reconstruction often arises from post-traumatic or iatrogenic causes, which usually implies a prior surgery or radiation therapy, making the surgical approach and operative field significantly more challenging for a minimally invasive procedure [4], [5], [7]. Likewise, the share of robotic surgery for the use of ileum interposition as a procedure requiring higher surgical skill is especially low with 13% in 2022.

Under the increasing health policy pressure to reduce both hospital LOS and health care costs in general from the mid-2000s in Germany, a decline in the number of days patients spend in the hospital can be observed for many different urological conditions and corresponding operative therapies [30]. This is also evident for the procedures examined in this study. However, there are significant differences in the average LOS for each. As shown above, the median LOS for robotic pyeloplasty was 5 d in 2022, compared with the significantly higher LOS for ureteral reconstruction, ranging from 11 d for partial ureteral replacement to 14 d for ileal interposition in 2022. These differences can initially be explained by the significantly varying levels of difficulty and complexity of the respective procedures. It can be assumed that a patient can be discharged a few days postoperatively after an uncomplicated robotic pyeloplasty, while after an extensive ureteral reconstruction with bowel segments, patients require a longer time for recovery and mobilization [10]. Additionally, especially in the early years following the introduction of robotic surgical systems, a certain positive selection of patients for this new technology is to be expected [31]. In general, when interpreting these results, it must be considered that there are significant differences in postoperative care of patients, particularly in discharge management, between Central European healthcare systems such as the German one and, for example, the American system, which demands significantly earlier postoperative discharge of patients [32]. Further, renal autotransplantation or implantation of an artificial ureter is an additional option for ureteral reconstruction. However, only few studies reported on these procedures, and our results showed that these are applied very rarely [33], [34].

### 4.3 Future trends

In the past years, reconstruction of the ureter using buccal grafts, especially in the case of mid to proximal longer strictures, has been much debated. Several authors describe the technique and promising success rates [11], [12]. However, there is still no code in the German version of the ICD-10-PCS codes (OPS codes) for this exact procedure. Therefore, we assessed the case numbers by combining codes for ureter reconstruction and extraction of a buccal graft. The resulting caseload is below expectation (*n* = 38) and does not reflect the magnitude of the discussion in the current literature. On the one hand, implementation of specific coding and thus appropriate remuneration for this surgical technique could encourage its increased use. On the other hand, there are only few reconstructive centers in Germany performing buccal grafts in upper tract procedures up to now. In exchange with a high-volume center for robotic urology, the advantages reported in literature, such as avoidance of intestinal complications and reduced LOS for stricture repair up to 6 cm, are reported to meet those findings published previously. It might therefore be a matter of time and experience for this technique to become more widespread in Germany. Nevertheless, the safe use of this procedure initially appears to be reserved for experienced hands in dealing with buccal grafts, for example, in urethral reconstructive surgery.

### 4.4 Limitations

The main limitations are imbedded in the nature of the data. Billing data are highly accurate, since reimbursement relies on it. However, detailed information on patient characteristics is not available and testing for coding errors is not possible. The verification of each data set is not possible since German anonymization requirements prevent identification from single patients or institutions from the DRG database. In addition, the anonymization provisions can lead, for some very specific questions with small case numbers, to partly censoring of the data. We sought to anticipate most of the relevant issues and adjust for these if possible. Given the extensive caseload numbers for these rare procedures, slight variances and small irregularities appear to be negligible. However, the principal risk of a systematic bias has to be kept in mind when interpreting the results. Further low mortality rates of pyeloplasties are associated with patient selection. Nevertheless, to the best of our knowledge, our study is the most comprehensive observation of trends and case numbers regarding reconstructive procedures of the ureter and renal pelvis in Germany.

## 5 Conclusions

This study showed an increasing trend toward minimally invasive pyeloplasty in recent years. For reconstructive procedures, the trend toward robotics was less significant. Despite being much debated, autotransplantation, artificial ureters, and buccal graft ureter reconstruction currently seem to be exceptional options and not routinely used alternatives. LOS decreased for all procedures and was shortest for the robotic approach.

  ***Author contributions*:** Luka Flegar had full access to all the data in the study and takes responsibility for the integrity of the data and the accuracy of the data analysis.

  *Study concept and design*: Flegar, Groeben.

*Acquisition of data*: Flegar, Eisenmenger, Groeben.

*Analysis and interpretation of data*: Flegar, Groeben, Moritz.

*Drafting of the manuscript*: Kipfer, Durmus, Karschuck, Aksoy, Reimold, Martin, Haak.

*Critical revision of the manuscript for important intellectual content*: Flegar, Groeben, Huber.

*Statistical analysis*: Koch.

*Obtaining funding*: None.

*Administrative, technical, or material support*: None.

*Supervision*: Flegar, Groeben, Huber.

*Other*: None.

  ***Financial disclosures:*** Luka Flegar certifies that all conflicts of interest, including specific financial interests and relationships and affiliations relevant to the subject matter or materials discussed in the manuscript (eg, employment/affiliation, grants or funding, consultancies, honoraria, stock ownership or options, expert testimony, royalties, or patents filed, received, or pending), are the following: Dr. Luka Flegar is a consultant for BK Medical and reports grants from Novartis and Astellas outside the presented work. Dr. Christer Groeben report fees from Janssen, Merck, and Bayer outside the presented work. Mrs. Nicole Eisenmenger is the founder and director of RI Innovation GmbH. Dr. Johannes Huber reports grants and nonfinancial support from Intuitive Surgical, Takeda, Janssen, Apogepha, and Coloplast outside the submitted work, and he is a member of the medical board of the Urological Foundation for Health. All other authors declare that there is no conflict of interest.

  ***Funding/Support and role of the sponsor*:** None.

  ***Data sharing statement*:** Data source: German research data center of the federal statistical office, DRG statistics 2006–2020, German “National Centre for Cancer Registry Data” (Robert Koch Institute, Berlin), own calculations. Data availability statement: German hospital quality reports are publicly accessible. The datasets generated and/or analyzed during the current study are available from the corresponding author on reasonable request.

  ***Ethics statement*:** This study was conducted in accordance with the Declaration of Helsinki in its latest version. For data protection reasons, within the quality reports, the diagnostics (ICD) data or intervention numbers (OPS) with a number of ≤3 does not indicate the actual number, but the number 1. All data used are anonymized, so no further ethics committee approval was required. This article does not contain any studies with animals performed by any of the authors. Further, a written informed consent was not needed.

## References

[1] Tyritzis S.I., Wiklund N.P. (2015). Ureteral strictures revisited…trying to see the light at the end of the tunnel: a comprehensive review. J Endourol.

[2] Roberts W.W., Cadeddu J.A., Micali S., Kavoussi L.R., Moore R.G. (1998). Ureteral stricture formation after removal of impacted calculi. J Urol.

[3] Tonyali S., Yilmaz M., Tzelves L. (2023). Predictors of ureteral strictures after retrograde ureteroscopic treatment of impacted ureteral stones: a systematic literature review. J Clin Med.

[4] Kranz J., Brandt A.S., Anheuser P., Reisch B., Steffens J., Roth S. (2017). Radiogene Harnleiterstrikturen: Mögliche Therapieoptionen. Urologe A.

[5] Lobo N., Kulkarni M., Hughes S., Nair R., Khan M.S., Thurairaja R. (2018). Urologic complications following pelvic radiotherapy. Urology.

[6] Chua M.E., Ming J.M., Kim J.K. (2021). Meta-analysis of retroperitoneal vs transperitoneal laparoscopic and robot-assisted pyeloplasty for the management of pelvi-ureteric junction obstruction. BJU Int.

[7] Bilotta A., Wiegand L.R., Heinsimer K.R. (2021). Ureteral reconstruction for complex strictures: a review of the current literature. Int Urol Nephrol.

[8] Knight R.B., Hudak S.J., Morey A.F. (2013). Strategies for open reconstruction of upper ureteral strictures. Urol Clin North Am.

[9] Light A., Karthikeyan S., Maruthan S., Elhage O., Danuser H., Dasgupta P. (2018). Peri-operative outcomes and complications after laparoscopic vs robot-assisted dismembered pyeloplasty: a systematic review and meta-analysis. BJU Int.

[10] Xiong S., Zhu W., Li X., Zhang P., Wang H., Li X. (2020). Intestinal interposition for complex ureteral reconstruction: a comprehensive review. Int J Urol.

[11] Heijkoop B., Kahokehr A.A. (2021). Buccal mucosal ureteroplasty for the management of ureteric strictures: a systematic review of the literature. Int J Urol.

[12] You Y., Gao X., Chai S. (2023). Oral mucosal graft ureteroplasty versus ileal ureteric replacement: a meta-analysis. BJU Int.

[13] Stühler V., Bedke J., Stenzl A. (2019). Rekonstruktionsmöglichkeiten des Harnleiters. Urologe A.

[14] Windsperger A.P., Duchene D.A. (2013). Robotic reconstruction of lower ureteral strictures. Urol Clin North Am.

[15] Yang K., Pang K.H., Fan S. (2023). Robotic ureteral reconstruction for benign ureteral strictures: a systematic review of surgical techniques, complications and outcomes: robotic ureteral reconstruction for ureteral strictures. BMC Urol.

[16] Zacharis A., Reimold P., Aksoy C. (2024). Trends in kidney transplantation and living donor nephrectomy in Germany: a total population analysis from 2006 to 2021. World J Urol.

[17] Hopf H.L., Bahler C.D., Sundaram C.P. (2016). Long-term outcomes of robot-assisted laparoscopic pyeloplasty for ureteropelvic junction obstruction. Urology.

[18] Moretto S., Gandi C., Bientinesi R. (2023). Robotic versus open pyeloplasty: perioperative and functional outcomes. J Clin Med.

[19] Mantica G., Ambrosini F., Parodi S., Tappero S., Terrone C. (2020). Comparison of safety, efficacy and outcomes of robot assisted laparoscopic pyeloplasty vs conventional laparoscopy. Res Rep Urol.

[20] Chow K., Adeyoju A.A. (2011). Section of Endourology of the British Association Of Urological Surgeons. National practice and outcomes of laparoscopic pyeloplasty in the United Kingdom. J Endourol.

[21] Braga L.H.P., Pace K., DeMaria J., Lorenzo A.J. (2009). Systematic review and meta-analysis of robotic-assisted versus conventional laparoscopic pyeloplasty for patients with ureteropelvic junction obstruction: effect on operative time, length of hospital stay, postoperative complications, and success rate. Eur Urol.

[22] Rodrigues Pessoa R., Urkmez A., Kukreja N., Baack K.J. (2020). Enhanced recovery after surgery review and urology applications in 2020. BJUI Compass.

[23] Grivas N., Zachos I., Georgiadis G., Karavitakis M., Tzortzis V., Mamoulakis C. (2022). Learning curves in laparoscopic and robot-assisted prostate surgery: a systematic search and review. World J Urol.

[24] Flegar L., Kraywinkel K., Zacharis A. (2022). Treatment trends for muscle-invasive bladder cancer in Germany from 2006 to 2019. World J Urol.

[25] Giri S.K., Murphy D., Costello A.J., Moon D.A. (2011). Laparoscopic pyeloplasty outcomes of elderly patients. J Endourol.

[26] Stockheim J., Andric M., Acciuffi S. (2022). Auswirkungen der COVID-19-Pandemie auf die robotische Viszeralchirurgie in Deutschland. Chirurgie (Heidelb).

[27] Sugand K., Aframian A., Park C., Sarraf K.M. (2022). Impact of COVID-19 on acute trauma and orthopaedic referrals and surgery in the UK during the first wave of the pandemic: a multicentre observational study from the COVid Emergency-Related Trauma and orthopaedics (COVERT) Collaborative. BMJ Open.

[28] David M, Weimann J, Bobbert P, Gehle P. Verschiebung elektiver Eingriffe: wie man priorisieren kann. Dtsch Ärztebl 2021;118:A-926/B-772.

[29] Groeben C., Koch R., Baunacke M. (2021). Entwicklung der operativen Uroonkologie in Deutschland – vergleichende Analysen aus populationsbasierten Daten. Urologe A.

[30] Herout R., Baunacke M., Flegar L. (2022). Upper tract urothelial carcinoma in Germany: epidemiological data and surgical treatment trends in a total population analysis from 2006 to 2019. World J Urol.

[31] Groeben C., Koch R., Baunacke M., Wirth M.P., Huber J. (2017). High volume is the key for improving in-hospital outcomes after radical prostatectomy: a total population analysis in Germany from 2006 to 2013. World J Urol.

[32] Ridic G., Gleason S., Ridic O. (2012). Comparisons of health care systems in the United States, Germany and Canada. Mater Sociomed.

[33] Joshi P., Lin J., Sura T. (2021). Kidney autotransplantation for treatment of ureteric obstruction: a case report and brief review of the literature. Case Rep Surg.

[34] Stoykov B., Kolev N., Dunev V., Mladenov V., Vanov A., Genov P. (2021). Subcutaneous nephrovesical bypass in a patient with advanced prostate cancer. Urol Case Rep.

