# Supplementary material for: Pyeloplasty and Ureteral Reconstruction Surgery Trends: A Total Population Analysis in Germany from 2006 to 2022
Source: Eur Urol Open Sci. 2024 Oct 24;70:116–23. doi: 10.1016/j.euros.2024.10.011 (PMC11541678; doi:10.1016/j.euros.2024.10.011)
Supplement: Supplementary Data 1 [file mmc1.docx]

**Supplementary Table 1.** Overview of the queried databases.

| **Data source** | Nationwide hospital billing database of the German Federal Statistical Office  (Destatis database) | German hospitals’ quality reports  (reimbursement.INFO tool) |
| --- | --- | --- |
| **Data details** | - Age and gender - Diagnosis code - Type of surgery and approach - Hospital characteristics (teaching status, size, annual surgery caseload, approaches for surgery) - Patients age ≥18 | - Age and gender - Type of surgery - Hospital characteristics (teaching status, annual surgery caseload) - Geographical localization of respective hospitals - Patients age ≥20 |
| **Data query option** | - Combination of OPS- and ICD- code possible | - Only OPS- or ICD-code |
| **Number of patients** | 34975 | 34875 |
| **Proportion of the country** | 100% | 100% |
| **Included years** | 2006 - 2022 | 2006, 2008, 2010, and 2012-2022 |

**Supplementary Fig. 1.** Age distribution in 2022 for adult patients receiving pyeloplasty in Germany.
